# Systematic Phosphorus‐Driven Structural and Field Engineering of n‐a‐Si:H for Flexible n‐a‐Si:H/Te Near‐Infrared Photodetectors

**DOI:** 10.1002/advs.76332

**Published:** 2026-07-06

**Authors:** Kyeong‐jin Hyun, Soo‐Won Choi, Byeongjin Park, Hee‐Won Jang, Jongwon Yoon, Yonghun Kim, Woon Ik Park, Jung‐Dae Kwon

**Affiliations:** ^1^ Energy & Environment Materials Research Division Korea Institute of Materials Science Changwon Gyeongnam Republic of Korea; ^2^ Department of Materials Science and Engineering Pukyong National University Busan Republic of Korea; ^3^ Department of Materials Science and Engineering Ulsan National Institute of Science and Technology (UNIST) Ulsan Republic of Korea; ^4^ Department of Materials Science and Engineering Pusan National University Busan Republic of Korea

**Keywords:** field engineering, flexible, hydrogenated amorphous silicon (a‐Si:H), near‐infrared, optoelectronics, phosphorus doping, photodetectors

## Abstract

Flexible near‐infrared (NIR) photodetectors are promising for optical communication, imaging, and wearable sensing, yet simultaneously achieving high responsivity, low dark current, and mechanical robustness remains challenging. Here, we report a flexible *n*‐type hydrogenated amorphous silicon/tellurium (n‐a‐Si:H/Te) heterojunction photodiode enabled by systematic control of the phosphine‐to‐silane dilution ratio and the introduction of a front‐surface‐field (FSF) layer. At the optimized phosphine dilution (P ratio = 25%), the n‐a‐Si:H film exhibits reduced defect density and improved structural ordering, forming an electronically coherent heterojunction with crystalline Te. The resulting device shows pronounced diode rectification and a strong photoresponse at 1050 nm, driven by efficient Te absorption and built‐in‐field‐assisted carrier separation. Furthermore, inserting a 10‐nm‐thick heavily doped n‐a‐Si:H FSF layer between the transparent conductive oxide and the active layer enhances band bending and suppresses interfacial recombination, leading to 5.1‐ and 2.6‐fold improvements in responsivity and detectivity, respectively. External quantum efficiency analysis confirms that the performance enhancement originates from electrical field modulation rather than optical effects. The FSF‐engineered device exhibits broadband operation (400–1600 nm) and maintains over 90% of its initial responsivity after 4000 bending cycles, demonstrating a robust strategy for high‐performance flexible NIR optoelectronics.

## Graphical abstract

Our research elucidates the systematic evolution of the microstructure and optoelectronic properties of n‐a‐Si:H governed by the phosphine dilution ratio. Optimally phosphorus‐doped n‐a‐Si:H is integrated with Te to form a flexible heterojunction, enabling high‐performance near‐infrared photodetectors. Moreover, a heavily doped n‐a‐Si:H front‐surface‐field layer inserted between the TCO and active layer facilitates effective band engineering and significantly enhances the NIR photoresponse.

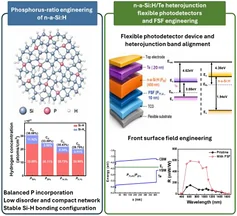

## 1 Introduction

Flexible near‐infrared (NIR) photodetectors have considerable potential for a wide range of emerging applications, including artificial intelligence, optical communication, medical diagnostics, wearable electronics, and autonomous systems [1, 2, 3]. However, integrating such devices with next‐generation wearable platforms requires the development of flexible photodetectors with high mechanical durability, strong optical responsivity, and cost‐effective manufacturing, which remains a formidable challenge [4]. Most commercialized NIR photodetectors based on inorganic semiconductors, such as crystalline silicon (c‐Si), germanium, and indium gallium arsenide, exhibit excellent performance and reliability. Nevertheless, they have complex fabrication procedures, high‐temperature epitaxial growth, high production costs, and poor mechanical compliance owing to rigid substrates [5, 6, 7, 8, 9]. In recent years, organic materials and quantum‐dot‐based photodetectors have attracted attention because of their intrinsic flexibility and tunable optoelectronic properties. However, their instability under ambient humidity and oxygen conditions and environmental concerns related to toxic heavy metals, such as lead, continue to hinder their practical deployment [10, 11].

Te is a chalcogen‐based narrow‐bandgap semiconductor and is an emerging alternative that bridges the gap between conventional inorganic and organic photodetectors [12, 13]. Transition‐metal dichalcogenides, such as molybdenum disulfide and bismuth telluride, typically exhibit limited carrier mobility and interfacial trap states. In contrast, Te has intrinsically high hole mobility and excellent crystallinity even at room temperature [14, 15, 16, 17]. Additionally, its thickness‐dependent direct bandgap (0.3–1.0 eV) enables broadband detection in the visible‐to‐infrared region [18]. Te is composed of helical atomic chains bound by van der Waals forces, which provide anisotropic charge transport and intrinsic mechanical flexibility that are suitable for strain‐tolerant devices [19, 20]. Moreover, its high optical absorption coefficient, ambient chemical stability, and single‐element composition effectively suppress the formation of secondary phases and point defects [21, 22]. Therefore, Te has emerged as a promising p‐type semiconductor for NIR photodetection because of its efficient hole transport, strong optical absorption, and excellent mechanical compliance [23].

A p–n heterojunction, specifically a diode‐type structure that separates photogenerated carriers, must be formed for the effective operation of Te‐based photodetectors [24, 25]. Although Te possesses high hole mobility and a narrow direct bandgap, its intrinsic *p*‐type conduction restricts electron extraction when used in isolation [26]. Therefore, coupling Te with an *n*‐type semiconductor that provides stable band alignment and low interfacial recombination is crucial for achieving efficient photoresponse in diode configurations. From this perspective, inorganic thin‐film semiconductors are particularly advantageous because of their superior chemical robustness, mechanical durability, and complementary metal–oxide semiconductor (CMOS) process compatibility compared with their organic counterparts [27, 28]. Among these, hydrogenated amorphous silicon (a‐Si:H) is well‐known for its ability to be uniformly deposited over large areas at temperatures below 250°C via plasma‐enhanced chemical vapor deposition (PECVD), precise tuning of carrier concentration and dark current through doping‐gas optimization, nontoxicity, and structural simplicity [29].

Conventional a‐Si:H typically exhibits a low carrier mobility, high defect density, and interfacial instability, which can induce band discontinuities and severe carrier recombination when interfaced with Te [30]. These limitations can be overcome by engineering the microstructure and doping behavior of a‐Si:H to regulate hydrogen bonding configurations and defect formation kinetics [31, 32, 33]. Controlled phosphine (PH_3_) incorporation not only modifies the concentration of Si–H and Si–H_2_ bonds but also refines the amorphous network ordering, thereby changing the local electronic potential landscape. This synergistic structural–electronic modulation reduces defect‐assisted recombination, enhances hydrogen passivation efficiency, and stabilizes the interface with Te by minimizing band discontinuities and trap‐assisted tunneling. Such improvements facilitate efficient electron extraction and interfacial charge transport, thus providing a basis for developing low‐temperature, large‐area, and flexible Te/a‐Si:H photodiodes with stable NIR photoresponse.

In this study, we develop a flexible broadband photodiode based on a Te and *n*‐type a‐Si:H (n‐a‐Si:H) heterojunction for NIR detection. By systematically tuning the PH_3_ dilution ratio (*P* ratio; ratio between silane (SiH_4_) and PH_3_) during the PECVD growth of n‐a‐Si:H, we elucidate the correlations between the doping concentration, structural disorder, and charge transport behavior. An optimized n‐a‐Si:H film is used to create a dense amorphous network with minimized microvoids and a low Urbach energy, resulting in weak dark current and high carrier extraction efficiency. To enhance the internal electric field and reduce interfacial recombination, a 10‐nm‐thick front‐surface‐field (FSF) layer with higher phosphorus doping is incorporated between the transparent conductive oxide (TCO) and n‐a‐Si:H layer. The Te/n‐a‐Si:H photodiode with the FSF layer exhibits a high responsivity of 0.48 A/W, detectivity of 2.47 × 10^10^ Jones at 1050 nm, and rectification ratio above 10^3^. Moreover, the device maintains over 90% of its initial responsivity even after 4000 bending cycles with a bending radius of 5 mm, thus demonstrating excellent mechanical robustness. These results highlight the potential of Te/n‐a‐Si:H heterojunctions as mechanically stable and CMOS‐compatible inorganic platforms for flexible and wearable broadband optoelectronics.

## 2 Experimental Materials and Methods

### 2.1 Fabrication of Flexible n‐a‐Si:H/Te Heterojunction Photodetector

Figure 1 shows the fabrication process of the flexible n‐a‐Si:H/Te heterojunction NIR photodetector. Glass substrates (50 × 50 mm^2^, thickness: 1.1 mm) were sequentially cleaned via ultrasonic treatment in acetone and 2‐propanol for 10 min, followed by nitrogen blow‐ drying and thermal drying at 100°C. Subsequently, 20‐µm‐thick colorless polyimide (CPI) films were synthesized on the glass substrate using the method described in our previous study [34]. Indium tin oxide (In_2_O_3_:SnO_2_ = 90:10 wt.%) and aluminum‐doped zinc oxide (ZnO:Al_2_O_3_ = 99:1 wt.%) were deposited with thicknesses of 100 and 20 nm, respectively, via direct‐current pulsed magnetron sputtering (Figure 1a). Subsequently, 10‐nm‐thick FSF and 400‐nm‐thick n‐a‐Si:H films were grown on the TCO‐coated substrates via PECVD at 250°C. During deposition, hydrogen (H_2_), SiH_4_, and PH_3_ (diluted in 99% H_2_) gases were used as precursors under a radio frequency (RF) excitation of 13.56 MHz and 50 W. The detailed deposition parameters are listed in Table 1. Thereafter, a positive photoresist (PR) was spin‐coated on the n‐a‐Si:H films, which were exposed to ultraviolet (UV) light and developed to define an active area of 0.0001 cm^2^. A 20‐nm‐thick Te film was deposited at room temperature (25°C) on the PR‐patterned substrates using RF magnetron sputtering at a base pressure of 5.0 × 10^−6^ Torr, working pressure of 4.8 mTorr, and RF power of 20 W. Finally, Ni top electrodes were formed via electron‐beam evaporation followed by an acetone‐based PR lift‐off process. The optical top‐view image of the completed device is shown in Figure 1h.

**FIGURE 1:**
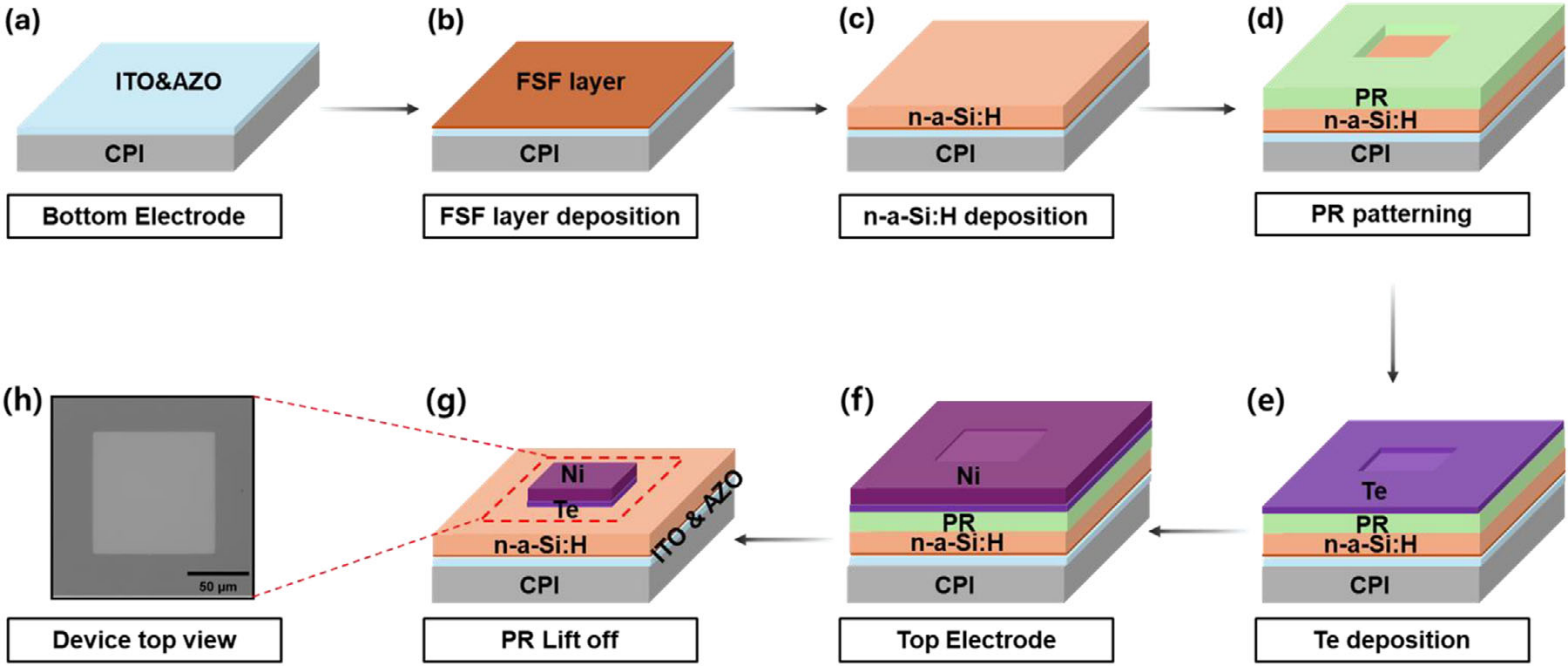
Schematic illustration of the fabrication process for the flexible n‐a‐Si:H/Te heterojunction near‐infrared (NIR) photodetector. (a) Sequential deposition of the indium tin oxide/aluminum‐doped zinc oxide (ITO/AZO) transparent conductive oxide (TCO) bilayer on a colorless polyimide (CPI) substrate; (b) formation of a front‐surface field (FSF) layer via plasma‐enhanced chemical vapor deposition (PECVD); (c) subsequent deposition of the n‐a‐Si:H layer onto the FSF‐coated substrate; (d) photolithographic definition of the active area using a positive photoresist (PR); (e) deposition of a Te film onto the patterned PR mask; (f) electron‐beam evaporation of Ni top electrodes, followed by (g) lift‐off processing to complete device patterning; (h) optical microscopy image of the fully fabricated flexible photodetector.

**TABLE 1:** Detailed PECVD deposition parameters of n‐a‐Si:H and FSF.

	Plasma Power [W]	Working Pressure [mTorr]	SiH_4_:H_2_	SiH_4_:PH_3_	Thickness [nm]
n‐a‐Si:H	50	600	1:4	1:1, 3:1, 6:1, 10:1	400
FSF	50	600	1:4	3:5, 2:5, 1:5	10

### 2.2 Characterization

For material characterization, 400‐nm‐thick n‐a‐Si:H and 20‐nm‐thick Te films were prepared on glass substrates. The film thickness and complex dielectric function (ε_1_ and ε_2_) of n‐a‐Si:H were evaluated using spectroscopic ellipsometry (SE MG‐1000, NanoView). The bonding configurations of n‐a‐Si:H were analyzed using Fourier‐transform infrared (FTIR) spectroscopy (Nicolet iS 10, Thermo Fisher Scientific Inc.), and the microstructural characteristics of Te and n‐a‐Si:H were examined using Raman spectroscopy (XperRAM‐CS, NANOBASE). The optical absorption characteristics and bandgap energies (E_g_) were determined using a UV–vis–NIR spectrophotometer (Cary 5000, Varian) using Tauc plot analysis. The work function and valence‐band maximum (VBM) were determined using UV and X‐ray photoelectron spectroscopy (UPS, XPS; AXIS SUPRA, KRATOS Analytical Ltd.). Additionally, the chemical bonding states of Te and n‐a‐Si:H were investigated via XPS, and the crystallographic orientation of Te was characterized using X‐ray diffraction (XRD; EMPYREAN, Malvern Panalytical). The surface morphology of the flexible photodetector was observed using an optical microscope (OPTIC‐CX40M‐TR, Optic Korea). The layer configuration and cross‐sectional morphology were analyzed using field‐emission transmission electron microscopy (TEM; JEM‐2100F HR, Jeol Ltd.) with focused‐ion‐beam (Helios 5 UX, Thermo Fisher) specimens. The dark conductivity of n‐a‐Si:H and the optoelectronic characteristics of the flexible photodiode were measured using a probe station (MS‐TECH) equipped with a Keithley 4200A source meter. For optoelectronic testing, the devices were placed in a dark‐shielded chamber (MS‐Tech) integrated with an LED controller (BioLED, Mightex) and a 1050‐nm light source (BLS‐LCS‐0470‐03‐22, Mightex). Mechanical flexibility was evaluated through cyclic bending tests using a bending stage (M‐433, Newport) with various radii of curvature (5, 10, 15, and 20 mm).

## 3 Results and Discussion

The structural characteristics of the n‐a‐Si:H films are primarily governed by the bonding configurations between Si, H, and P atoms within the amorphous network. The intrinsic amorphous matrix can be modulated by the structural state of hydrogen‐passivated Si atoms and the internal strain induced by heteroatomic dopants. FTIR spectroscopy is used to systematically examine the evolution of the atomic‐scale bonding environment with the *P* ratio (Figure 2a). The n‐a‐Si:H films are denoted as *P*_x%_, where *P*_x%_ represents the phosphine fraction defined as PH_3_/(SiH_4_ + PH_3_) × 100%. In the FTIR spectra, the absorption bands centered near 2000 and 2100 cm^−1^ correspond to the stretching vibrations of the Si–H and Si–H_2_ bonds, respectively [35]. The dihydride mode around 2100 cm^−1^ is highly sensitive to the concentration of hydrogen bonded within microvoids and the strain induced by heteroatoms [36, 37, 38]. To quantify these parameters, each spectrum is deconvoluted into Si–H and Si–H_2_ components. As the *P* ratio decreases from 50% to 25%, the integrated area of the Si–H_2_ peak decreases, indicating a reduction in the microvoid‐related dihydride bonds and a densification of the amorphous network. However, further reduction in the *P* ratio to 14.3% and 9.1% results in a significant increase in the Si–H_2_ component. This is attributed to the combined effects of the diminished hydrogen contribution from the PH_3_ precursor and the intensified flux of SiH_x_ radicals generated from the silane plasma. Such an imbalance in the H/Si ratio leads to the partial depassivation of dangling Si bonds and reformation of Si–H_2_ configurations within the matrix. The microstructure factor (R^*^ = A_2100_/(A_2000_ + A_2100_)) is used to evaluate the relative dihydride content. The calculated values of R^*^ are 9.9%, 5.7%, 9.7%, and 8.0% for *P*_50%_, *P*_25%_, *P*_14.3%_, and *P*_9.1%_, respectively. To further quantify the hydrogen‐bonding configurations, the hydrogen concentrations associated with monohydride‐ and higher‐order hydride‐related states are estimated from the integrated absorbance of the stretching modes [36]. Distinct proportionality constants are applied to the 2000 and 2100 cm^−1^ bands, and the total hydrogen concentration is obtained from the sum of the two contributions (Figure S1). The resulting analysis shows that, although the total hydrogen content increases with increasing phosphorus incorporation, the *P*_25%_ film exhibits the lowest fraction of higher‐order hydride‐related bonding, indicating the most electronically stable hydrogen‐bonding environment among the compared n‐a‐Si:H films. These results indicate that the *P*_25%_ film has the most compact and strain‐relieved amorphous matrix and suggest that a moderate PH_3_ dilution optimizes the hydrogen‐bonding configuration, thereby enhancing the structural stability of the n‐a‐Si:H network.

**FIGURE 2:**
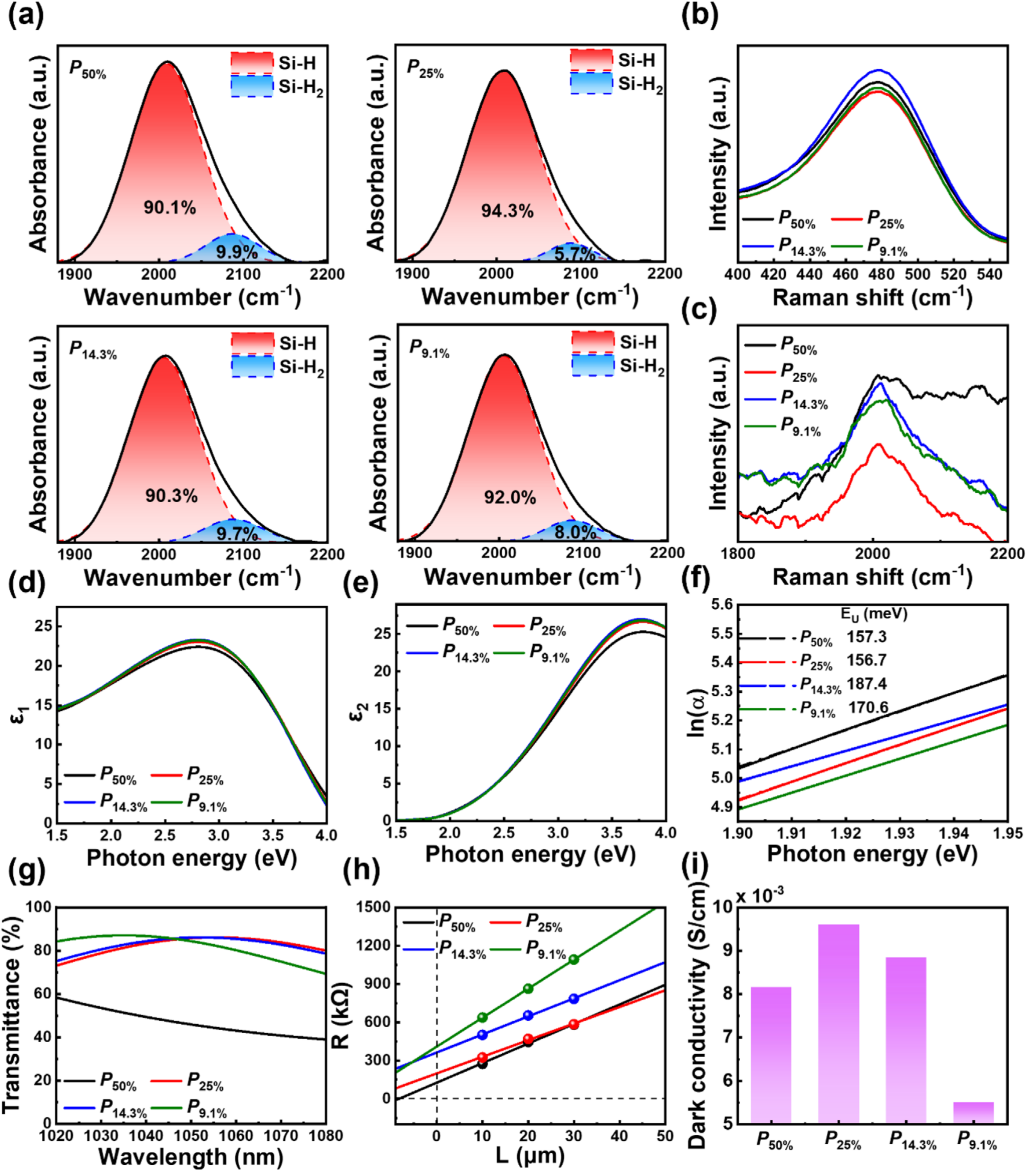
Material characteristics of n‐a‐Si:H films synthesized with varying phosphine‐to‐silane gas flow ratios. (a) Fourier‐transform infrared (FTIR) absorption spectra with Si─H bonding configuration analysis; (b, c) Raman spectra in the wavenumber ranges of 400–500 and 1800–2200 cm^−1^, respectively; (d, e) dielectric function spectra showing the real (ε_1_) and imaginary (ε_2_) parts obtained by spectroscopic ellipsometry, respectively; (f) logarithmic absorption coefficient spectra used to determine the Urbach energy (E_U_); (g) optical transmittance spectra; (h) transmission line method (TLM) results for extracting sheet resistance; (i) dark conductivity of the n‐a‐Si:H films.

Figure 2b,c shows the Raman spectra of n‐a‐Si:H films in two distinct wavenumber ranges: 400–550 cm^−1^, which reflects the structural phase of the matrix, and 1800–2200 cm^−1^, which provides insight into the local hydrogen‐related microstructure [39]. All films exhibit a broad amorphous band centered at 480 cm^−1^ without crystalline features near 510 or 520 cm^−1^, indicating the absence of crystallization even at high PH_3_ concentrations. This confirms that the hydrogen supplied by the PH_3_ precursor does not induce atomic ordering but stabilizes the amorphous network. Interestingly, the film with the highest *P* ratio (*P*_50%_) exhibits a broadened and intensified Raman background above 2000 cm^−1^, signifying a larger degree of structural disorder and bond‐angle distortion within the hydrogen network [40]. This can be ascribed to excessive phosphorus incorporation, which induces local strain and enhances dihydride bond‐related configurations through defect formation. This behavior agrees well with the FTIR results shown in Figure 2a. R^*^ is the highest for *P*_50%_, indicating a looser and void‐rich amorphous matrix under heavy doping conditions. Figure 2d,e present the real (ε_1_) and imaginary (ε_2_) components of the dielectric function for n‐type a‐Si:H films deposited at various *P* ratios, as obtained from spectroscopic ellipsometry. All spectra exhibit the typical optical response of a‐Si:H, featuring a distinct maximum for ε_1_ around 2.7 eV and a gradual increase in ε_2_ toward higher photon energies, which corresponds to band‐to‐band transitions near the optical absorption edge [41]. As the *P* ratio decreases from 50% to 9.1%, ε_1_ and ε_2_ slightly increase across the entire spectral range, indicating enhanced film density and electronic polarizability [42]. This behavior implies that moderate PH_3_ dilution promotes a more tightly bonded amorphous network with increased dielectric polarization rather than subgap defects. The simultaneous increase in ε_1_ and ε_2_ suggests that the local bonding environment becomes more coherent and optically compact owing to improved Si–Si and Si–H network connectivity. In contrast, the heavily doped *P*_50%_ film exhibits a slightly reduced value of ε_1_, implying structural disorder and strain‐induced fluctuations in the electronic potential. The lowered ε_2_ of the heavily doped *P*_50%_ film is attributed to weakened interband transition strength arising from structural dilution of the amorphous network and reduced electronic polarizability under excessive phosphorus incorporation. This interpretation is consistent with the degraded conductivity of the *P*_50%_ film, implying that the same underlying disorder responsible for the reduced dielectric response also enhances defect‐assisted carrier scattering and degrades transport [43, 44]. These results confirm that optimal *P* ratio (25%) yields the densest and most optically stable a‐Si:H matrix. To quantitatively evaluate the degree of structural disorder in the amorphous network, the Urbach energy (E_U_) is obtained from the exponential region of the logarithmic absorption coefficient (α), based on the spectra of α shown in Figure S2 (Figure 2f). E_U_ represents the width of the band‐tail states near the optical band edge, which are governed by structural defects and local disorder. Therefore, it is a reliable indicator of thermally activated and field‐assisted carrier losses in amorphous semiconductors [45, 46]. E_U_ slightly decreases as the *P* ratio decreases from 50% to 25%, implying a narrowing of the band‐tail distribution and an improvement in structural uniformity. However, the E_U_ increases as the *P* ratio further decreases from 14.3% to 9.1%. This indicates that excessive SiH_4_ dilution reintroduces bond‐angle disorder and defect‐related tail states owing to an imbalance between hydrogen supply and SiH_x_ radical flux. This trend is consistent with the FTIR analysis results shown in Figure 2a, thus confirming that moderate PH_3_ dilution (*P*_25%_) yields a denser and more structurally stable n‐a‐Si:H matrix with minimal subgap disorder.

As illustrated in Figure 1, the n‐a‐Si:H layer functions as the front window layer in the photodetector device and allows incident light to reach the underlying Te absorber, which exhibits strong absorption in the NIR region. To facilitate efficient NIR photon coupling in the Te layer, the n‐a‐Si:H film must exhibit high optical transmittance at a wavelength of 1050 nm. Figure 2g shows the transmittance spectra of the n‐a‐Si:H films in the NIR region centered at 1050 nm. Although the 400‐nm‐thick films show periodic interference fringes originating from multiple reflections within the thin‐film stack, a clear dependence on the *P* ratio is observed. Films with lower *P* ratios exhibit higher transmittance in the 1000–1100 nm region, indicating reduced subgap absorption and improved optical transparency. This enhancement is attributed to the denser and less‐disordered amorphous matrix, as confirmed by FTIR and Raman analyses. Consequently, the n‐a‐Si:H film with the optimum *P* ratio ensures efficient light transmission toward the Te layer, thereby enhancing the overall NIR photoresponse of the device. As the structural disorder and *P* ratio of the n‐a‐Si:H films strongly influence their electrical transport properties, the sheet and contact resistances are analyzed using the transfer‐line method (TLM), and the results are presented in Figure 2h. The TLM relationship is expressed as $R_{total}=2R_{C}+\frac{R_{S}}{W}\cdot L$, where 𝑅*_total_* is the measured resistance between two contacts separated by distance 𝐿, 𝑅*_C_* is the contact resistance, 𝑅𝑠 is the sheet resistance, and *W* is the electrode width. The current–voltage (*I*–*V*) characteristics measured at different electrode spacings for the TLM analysis are presented in Figure S3. As the *P* ratio decreases from 50% to 25%, the slope of the TLM line flattens, indicating a reduction in the sheet resistance. The slope increases again as the *P* ratio decreases further, suggesting a loss of electrical conduction paths due to insufficient doping or enhanced defect scattering. When these results are converted into dark conductivity (Figure 2i), the *P*_25%_ film exhibits the highest conductivity, whereas the heavily doped (*P*_50%_) and excessively SiH_4_ diluted (*P*_9.1%_) films show inferior transport characteristics. These results confirm that moderate PH_3_ dilution optimizes the carrier concentration and film microstructure. Excessive PH_3_ induces local strain and defect‐assisted scattering, whereas excessive SiH_4_ dilution reduces donor incorporation and passivation efficiency. Consequently, a *P* ratio of approximately 25% provides the best trade‐off between doping efficiency and structural stability, resulting in superior electronic transport in n‐a‐Si:H films.

The heterojunction photodiode based on Te thin films primarily absorbs light in the NIR region, where the photoresponse critically depends on the intrinsic optoelectronic properties of Te that govern electron–hole pair generation. Te thin films can undergo oxidation more readily compared with their bulk counterparts, which leads to significant variations in the band structure. Hence, it is crucial to accurately identify the chemical state of Te in the as‐deposited film. Therefore, XPS analysis is performed to investigate the chemical bonding states of Te (Figure 3a). The Te 3d_5/2_ core‐level spectrum exhibits two distinct peaks at 573.0 and 576.1 eV corresponding to the Te─Te bonding state of Te^0^ and oxidized Te^4+^ species, respectively [47]. Although Te is generally prone to surface oxidation, the dominance of the Te component suggests that the deposited Te film retains its metallic bonding characteristics with minimal surface oxide formation. The crystalline structure and phase composition of the Te thin film are examined using Raman spectroscopy (Figure 3b). Three prominent peaks are observed at 93, 122.3, and 140.3 cm^−1^, which correspond to the Raman‐active modes of crystalline trigonal Te [48, 49, 50]. The strongest peak at 122.3 cm^−1^ is assigned to the A_1_ symmetric stretching mode, which originates from the vibration of atoms along the helical Te chains. The 93 cm^−1^ peak is attributed to the E^1^ shear vibrations between adjacent chains, while the 140.3 cm^−1^ peak corresponds to the E^2^ asymmetric stretching or bending modes within the chains. These results confirm that the deposited Te film contains a well‐defined trigonal crystalline phase composed of helically arranged Te atoms, which is consistent with the intrinsic lattice vibrations of bulk t‐Te. The crystal structure of the Te film is further investigated using XRD, and the results are shown in Figure 3c. The diffraction peaks observed at 2θ = 27.6°, 38.3°, and 40.5° correspond to the (100), (101), and (110) planes, respectively, which are in excellent agreement with the hexagonal (trigonal) Te phase (JCPDS No. 36–1452) [51]. Additionally, weaker reflections are detected in the (111) and (201) planes, which confirm the polycrystalline nature of the film. The dominance of the (100) reflection indicates a preferred orientation along the (100) plane, which is characteristic of Te films grown through sputtering methods. This preferential orientation suggests that helical Te chains, which are parallel to the *c*‐axis in the trigonal lattice, are coherently arranged along the film‐growth direction. Combined with the Raman analysis, these results confirm that the deposited Te film has a well‐crystallized trigonal structure with strong lattice ordering and minimal amorphous components.

**FIGURE 3:**
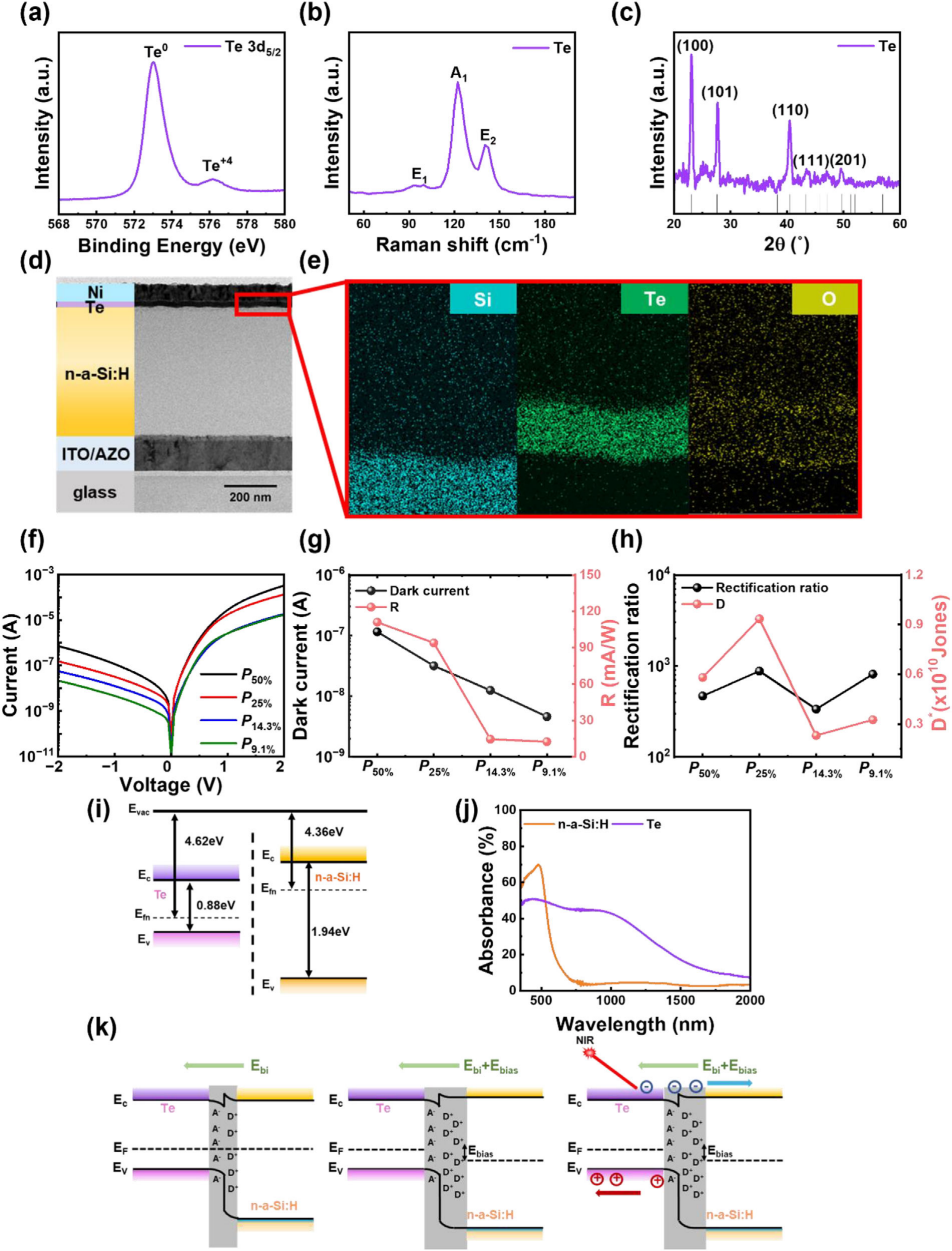
Material properties of the Te film and performance evaluation of the n‐a‐Si:H/Te heterojunction photodiode. (a) X‐ray photoelectron spectroscopy (XPS) spectrum of Te highlighting the Te 3d_5/2_ core level; (b) Raman spectrum and (c) X‐ray diffraction (XRD) pattern of Te confirming its crystalline structure. (d) Cross‐sectional transmission electron microscopy (TEM) image of the flexible n‐a‐Si:H/Te heterojunction device; (e) TEM‐energy dispersive X‐ray spectroscopy (EDS) elemental mapping at the n‐a‐Si:H/Te interface showing Si (cyan), Te (green), and O (yellow). Electrical characteristics of photodiodes with different phosphine‐to‐silane (P) ratios: (f) current–voltage (*I*–*V*) curves under 1050 nm light illumination, (g) dark current and responsivity, and (h) rectification ratio and detectivity. (i) Energy band diagram of Te and optimized n‐a‐Si:H heterojunction; (j) optical absorption spectra of n‐a‐Si:H and Te films; (k) schematic illustration of the device operating mechanism under reverse bias, showing carrier transport driven by the built‐in electric field.

The results of the chemical and structural analyses (Figure 3a–c) show that the Te film with a well‐crystallized trigonal phase and minimal oxidation provides a robust structural foundation for fabricating a heterojunction photodiode with n‐a‐Si:H. Cross‐sectional TEM is utilized to directly visualize the interfacial morphology and confirm the physical integrity of the n‐a‐Si:H/Te heterojunction (Figure 3d). The device shows a well‐organized multilayer architecture consisting of glass/TCO/n‐a‐Si:H/Te/Ni, where each layer is distinctly resolved without interdiffusion or void formation. The Te layer exhibits a dense crystalline texture and maintains a sharply defined boundary with the n‐a‐Si:H layer, thus confirming the formation of a structurally coherent and electronically favorable junction between the amorphous and crystalline phases. Such an abrupt and uniform interface is crucial for facilitating efficient carrier separation and transport in the photodiode, thereby minimizing recombination losses under NIR illumination. These structural observations are consistent with the XRD and Raman analyses, demonstrating that the trigonal Te lattice preserves its crystallographic order even after heterojunction formation. Elemental mapping is performed using energy‐dispersive spectroscopy (EDS) to further examine the compositional uniformity across the junction (Figure 3e). The spatially resolved maps distinctly reveal the distributions of Si (cyan), Te (green), and O (yellow), thus confirming a chemically abrupt n‐a‐Si:H/Te interface. The weak oxygen signal localized near the Te surface region is attributed to slight surface oxidation. This confirms that the fabricated n‐a‐Si:H/Te heterojunction contains a structurally stable and chemically well‐defined interface, which is expected to facilitate efficient NIR photon absorption and carrier transport during photodiode operation.

To evaluate the optoelectronic performance of the fabricated n‐a‐Si:H/Te heterojunction photodiodes, the *I*–*V* characteristics are measured under 1050‐nm illumination at an optical power of 1 mW (Figure 3f). As shown by the *I*–*V* curves in Figure S4, the fabricated device exhibits distinct diode characteristics and a pronounced photocurrent response that proportionally increases with the illumination intensity, thus demonstrating the typical operation of a photodiode. All devices exhibit distinct rectifying behaviors, which confirm the formation of a diode‐type carrier transport pathway across the n‐a‐Si:H/Te interface. The photocurrent at −1 V is systematically reduced with a decrease of the *P* ratio, suggesting that the electrical conductivity and carrier density of the n‐a‐Si:H layer strongly affect the photocurrent magnitude. To quantitatively evaluate this trend, the dark current and responsivity (R) are obtained at −1 V (Figure 3g). R is defined as $R\;=\frac{I_{P}-I_{d}}{P_{\lambda }\times S}\;$, where I_P_ and I_D_ are the photocurrent and dark current, respectively, P_λ_ is the incident optical power density, and S is the active device area. As the P ratio decreases, the dark current is significantly suppressed owing to the reduced doping concentration and minimized tunneling leakage. Conversely, the R suffers a reduction, as the poor conductivity of the excessively underdoped n‐a‐Si:H layer limits photocarrier extraction efficiency. The rectification ratio and specific detectivity (D^*^) are calculated to further assess the diode characteristics, as shown in Figure 3h. These are defined as follows: rectification ratio = $\frac{I_{forward}}{I_{reverse}}$, $D^{*}=\frac{R\sqrt{S}}{\sqrt{2eI_{d}}}\;$, where e is the elementary charge [52, 53]. Among all the devices, the photodiode with the *P*_25%_ film exhibits the highest rectification ratio (∼8.8 × 10^2^) and D^*^ (∼9.34 × 10^9^ Jones), indicating superior carrier selectivity and low noise characteristics. The *P*‐ratio‐dependent device behavior is closely correlated to the intrinsic material properties of n‐a‐Si:H, as shown in Figure 2. At moderate PH_3_ dilution (*P*_25%_), reduced Si–H_2_ bonding and suppressed structural disorder yield a dense amorphous network with enhanced electronic uniformity. The moderately doped n‐a‐Si:H film minimizes interface recombination and ensures efficient carrier transport, leading to superior rectification and D^*^ in the *P*_25%_ photodiode. This indicates that moderate PH_3_ doping provides an optimal balance between leakage suppression and carrier transport, thus improving the NIR detection performance.

Figure 3i shows the energy band diagram of the optimized n‐a‐Si:H/Te heterojunction (*P*_25%_). According to XPS, UPS, and Tauc plot analyses (Figure S5), the work functions (ϕ) of Te and n‐a‐Si:H are approximately 4.62 and 4.36 eV, and their bandgap energies (E_g_) are 0.88 and 1.94 eV, respectively [54]. These differences generate band offsets that establish a built‐in electric field (E_bi_) across the junction, which enables the effective separation of photogenerated carriers. Electrons drift toward n‐a‐Si:H and holes toward Te, thus minimizing interfacial recombination and facilitating charge extraction. The optical absorption spectra (Figure 3j) derived from the transmittance and reflectance spectra shown in Figure S6 clearly reveal the complementary optical roles of the two layers. Te exhibits strong absorption in the NIR region, whereas n‐a‐Si:H primarily absorbs visible light owing to its wider bandgap. At 1050 nm, absorption is dominated by Te, which confirms that NIR photoexcitation predominantly occurs within the Te layer. Consequently, n‐a‐Si:H serves as an electron‐collecting and optically transparent window layer that facilitates carrier extraction rather than photon absorption. This is further supported by the *I*–*V* characteristics of the n‐a‐Si:H film in the dark and under 1050‐nm illumination, which clearly show the absence of photoresponse (Figure S7).

The operational mechanism of the heterojunction photodiode is illustrated in Figure 3k. The electronic parameters of n‐a‐Si:H films used for the quantitative band analysis were extracted from the UPS cutoff/Fermi‐edge regions, XPS valence band maximum positions, and optical bandgap values determined by Tauc analysis (Figure S8). The quantitative band analysis summarized in Table 2 and the AFORS‐HET band diagrams (Figure S9) show that the pre‐contact n‐a‐Si:H/Te junction exhibits a type‐I (straddling) band alignment over the investigated phosphorus range, while the interfacial energetics evolve systematically with phosphorus concentration. The detailed simulation parameters are listed in Table S1. In the equilibrium state, the interfacial band discontinuity and post‐contact electrostatic equilibration induce a E_bi_ and form a depletion region that governs carrier‐selective transport. As the phosphorus ratio increases, the conduction‐band offset with Te is reduced from −0.39 to −0.05 eV, whereas the valence‐band offset increases from 0.65 to 0.97 eV, indicating progressively facilitated electron transfer from Te to n‐a‐Si:H together with strengthened hole blocking. In addition, the decrease in E_C_−E_F_ from 0.60 to 0.43 eV suggests that increasing donor incorporation modifies the interfacial band bending after junction formation. Using the experimentally extracted electronic parameters as inputs, AFORS‐HET simulations of the post‐contact n‐a‐Si:H/Te junction show that the built‐in potential decreases from 0.410 V (*P*_9.1%_) to 0.234 V (*P*_50%_), while the average built‐in electric field decreases from 1.02 × 10^4^ to 5.86 × 10^3^ V/cm with increasing phosphorus concentration (Figure S10). The detailed input parameters used for the AFORS‐HET simulations are summarized in Table S1. This trend is consistent with the UPS‐derived work‐function shift of n‐a‐Si:H toward the Te value, which reduces the work‐function mismatch across the heterojunction. Therefore, less electrostatic band bending is required to achieve Fermi‐level alignment after junction formation at higher phosphorus concentrations. Under reverse bias, the total electric field (E_bi_+E_bias_) increases the depletion width and promotes carrier drift toward the respective electrodes. Under NIR illumination, photons absorbed in the Te layer generate electron‐hole pairs that are rapidly separated by the internal field and selectively collected through the corresponding contacts. This carrier‐selective extraction is most effective for the optimally doped n‐a‐Si:H layer, leading to the highest rectification ratio and detectivity. Although the *P*_50%_ film exhibits the smallest conduction‐band offset, the best device performance is achieved at *P*_25%_, suggesting that optimal photodetection is governed by the combined effects of interfacial energetics and film quality rather than by the offset magnitude alone. This is further supported by the contact‐resistance trend: although the contact resistance of n‐a‐Si:H decreases monotonically with increasing phosphorus concentration (Figure S11) and is lowest for *P*_50%_, the best photodiode performance is still obtained at *P*_25%_. Therefore, neither the offset magnitude nor the contact resistance alone governs the device behavior; rather, the optimum performance arises from the most favorable overall balance among heterojunction band alignment, sufficiently low contact resistance, and material quality.

**TABLE 2:** Summary of energy levels of Te and phosphorus‐doped n‐a‐Si:H films, along with the valence and conduction band offsets at the n‐a‐Si:H/Te heterojunction.

*P* ratio of n‐a‐Si:H	Work function (eV)	E_g_ (eV)	VBM (eV)	E_C_‐E_F_ (eV)	CBM (eV)	Valence band offset with Te (eV)	Conduction band offset with Te (eV)
Te	4.62	0.88	−4.88	0.62	−4.00	—	—
*P*_9.1%_	4.21	1.92	−5.53	0.6	−3.61	0.65	−0.39
*P*_14.3%_	4.26	1.91	−5.65	0.52	−3.74	0.77	−0.26
*P*_25%_	4.36	1.94	−5.85	0.45	−3.91	0.97	−0.09
*P*_50%_	4.38	1.90	−5.85	0.43	−3.95	0.97	−0.05

Although the flexible NIR photodetector based on the n‐a‐Si:H/Te heterojunction is successfully fabricated, it exhibits a relatively low value of R. To combat this, a stronger internal electric field can be introduced on the n side of the p–n junction to enhance band bending and thus facilitate efficient charge collection. Accordingly, we designed an n‐a‐Si:H FSF layer with increased phosphorus doping and inserted it between the TCO and 400‐nm‐thick n‐a‐Si:H layer to serve as a highly doped field‐enhancing region (10 nm thick). To investigate the phosphorus incorporation behavior at high *P* ratios, XPS is performed using the *P*_62.5%_, *P*_71.4%_, and *P*_83.3%_ films and the results are compared with the optimized pristine film (*P*_25%_). As shown in Figure 4a, the deconvoluted P 2p spectra show peaks at 134, 130, and 129 eV corresponding to the P–O_x_, P–Si (2p_1/2_), and P–Si (2p_3/2_) bonding states, respectively. The two P–Si components are separated by approximately 1 eV owing to the spin–orbit coupling of the P 2p orbital. This confirms the well‐resolved doublet structure associated with substitutional phosphorus bonding in the amorphous Si network. As the *P* ratio increases, the relative intensity of the oxygen‐related species (P–O_x_) decreases, whereas the P–Si doublet becomes more pronounced. This indicates an improved substitutional incorporation of phosphorus and a higher *n*‐type doping level in n‐a‐Si:H. This trend confirms that higher PH_3_ flow enhances *n*‐type doping and facilitates the formation of a highly conductive FSF layer that is suitable for strong field‐assisted carrier collection.

**FIGURE 4:**
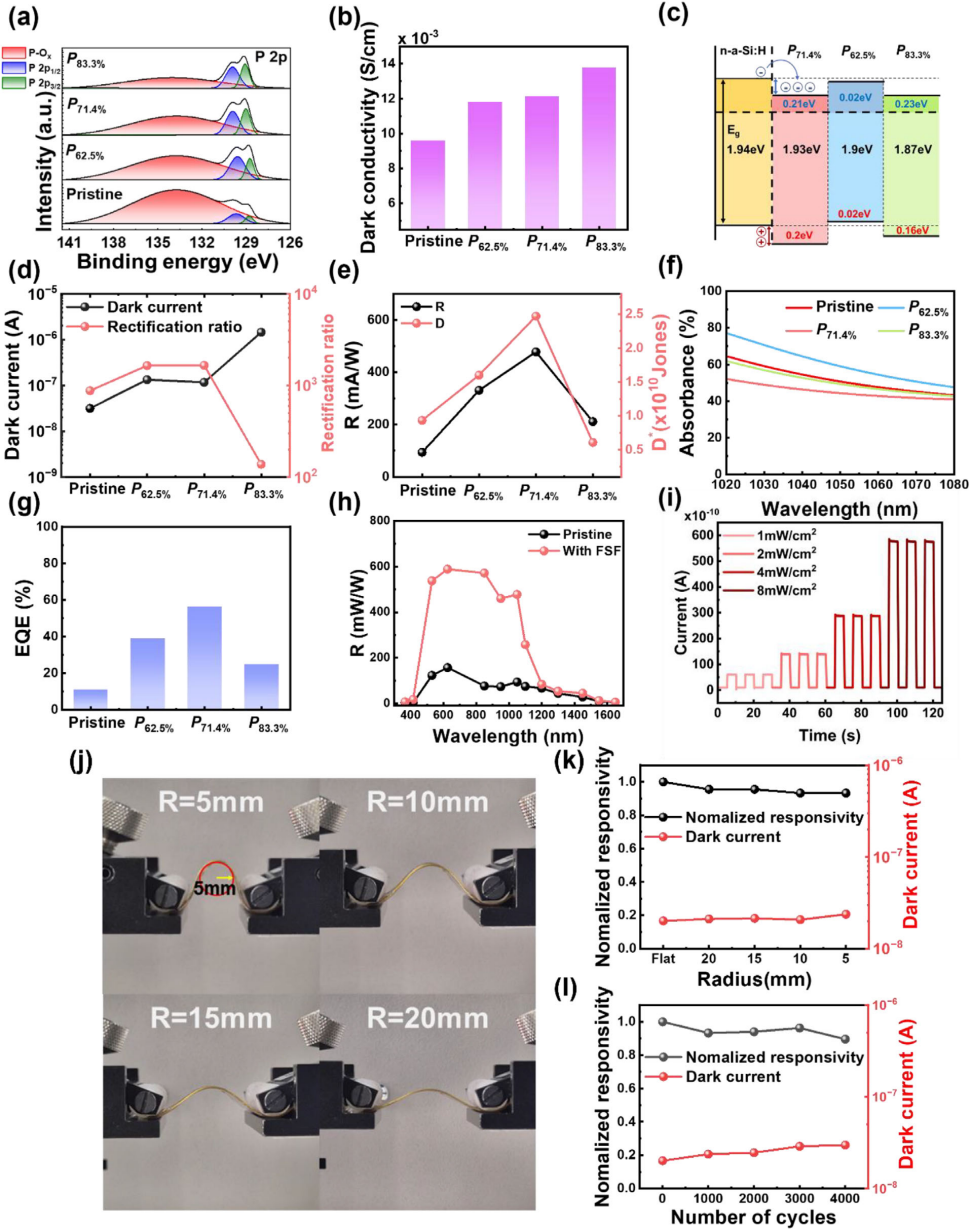
Characteristics of heavily doped n‐a‐Si:H films for designing a front‐surface field (FSF) layer and demonstration of the optimized device performance and flexibility. (a) XPS spectra of the P 2p core level for the FSF layer; (b) comparison of the dark conductivity of the FSF layer with the pristine condition; (c) energy band diagrams illustrating the calculated band alignment and barrier heights at the FSF/n‐a‐Si:H interface. Electrical characteristics of photodiodes incorporating the FSF layer: (d) dark current and rectification ratio, and (e) responsivity and detectivity under 1050 nm light illumination. (f) Optical absorbance spectra of the FSF and pristine n‐a‐Si:H films; (g) external quantum efficiency (EQE) of photodiodes with FSF incorporation under 1050 nm light illumination; (h) spectral responsivity of flexible photodetectors with and without the FSF layer; (i) time‐resolved photoresponse showing stable on/off switching under various light intensities. (j) Photographs of the flexible device under different bending radii (5–20 mm); normalized responsivity and dark current as functions of (k) bending radius after 1000 bending cycles and (l) repeated bending cycles at a 5 mm radius, respectively.

The dark conductivity of pristine n‐a‐Si:H and FSF‐introduced n‐a‐Si:H films (FSF 10 nm / pristine 400 nm) was evaluated to elucidate the impact of the FSF layer on the electrical transport characteristics (Figure 4b). Consistent with the monotonic phosphorus incorporation trend observed in the XPS analysis, a gradual enhancement in dark conductivity is observed with increasing phosphorus content in the FSF layer. This systematic improvement is attributed to the strengthened built‐in electric field induced by higher dopant incorporation within the ultra‐thin FSF layer. It is noteworthy that heavily doped n‐a‐Si:H films with high phosphorus ratios (*P*_62.5%_, *P*_71.4%_, and *P*_83.3%_) exhibit a degraded amorphous network, as evidenced by increased R^*^ values (Figure S12) and reduced ε_1_ and ε_2_ components (Figure S13) compared to the pristine film. However, when confined to an ultra‐thin thickness of 10 nm, the heavily doped n‐a‐Si:H layer effectively enhances the interfacial electric field without inducing significant microstructural deterioration, thereby functioning as a front surface field. Consequently, the ultra‐thin FSF layer compensates for intrinsic defect‐related limitations and facilitates electronic transport through improved dopant activation and carrier percolation within the amorphous matrix.

The functional behavior of the FSF layers is assessed using the energy‐band alignment between each FSF layer and pristine n‐a‐Si:H (Figure 4c). E_g_ and ϕ are determined for each film using Tauc plot, XPS, and UPS spectra (Figures S14 and S15). For the *P*_62.5%_ film, the conduction‐band offset (ΔE_C_) at the n‐a‐Si:H and FSF interface is only 0.02 eV, implying that band bending is insufficient for inducing a field effect. In contrast, the *P*_71.4%_ and *P*_83.3%_ films exhibit significantly larger offsets of 0.21 and 0.23 eV, respectively, thereby confirming the establishment of a pronounced energy barrier that can generate a strong field. Moreover, the valence‐band offset (ΔE_V_) of the *P*_71.4%_ film is 0.04 eV higher than that of the *P*_83.3%_ film, suggesting a more favorable band configuration that enhances electron selectivity while maintaining efficient hole blocking. Therefore, under the investigated conditions, the *P*_71.4%_ FSF layer is expected to provide the most balanced field strength and energy alignment for optimal carrier separation and collection in the n‐a‐Si:H/Te photodiode.

To investigate the performance of the device with the FSF layer, the dark current and rectification ratio of each flexible photodiode are measured at −1 V (Figure 4d). The dark current gradually increases with the increase in the *P* ratio, indicating the increase in the doping density and carrier concentration. In contrast, the rectification ratio increases up to a *P* ratio of 71.4% and then decreases at a *P* ratio of 83.3%. This indicates that excessive doping induces interface leakage and weakens barrier selectivity. To further clarify these tendencies, the values of R and D^*^ are evaluated for each flexible photodiode (Figure 4e). The trends for R and D^*^ agree with that for the rectification ratio, and the maximum values are obtained for the device with the *P*_71.4%_ FSF layer. The values of R and D^*^ for this optimized configuration are approximately 509% and 264% higher, respectively, than those for the pristine device, and the rectification ratio exceeds 10^3^. This demonstrates that the *P*_71.4%_ FSF layer effectively enhances band bending at the front junction, thereby improving carrier separation and collection, as predicted by the energy‐band analysis in Figure 4c.

The optical absorbance of the device, which is derived from the transmittance and reflectance spectra shown in Figure S16, is analyzed (Figure 4f) to accurately determine whether the observed improvement originates from electrical or optical effects. Interestingly, the *P*_71.4%_ device shows a relatively lower absorbance compared with the other devices despite having the best performance. This suggests that the efficiency is not governed by optical absorption but by electrical improvements induced by the formation of a FSF at the TCO/n‐a‐Si:H interface. The FSF strengthens upward band bending near the front surface, thus promoting stronger internal fields and more effective electron extraction from the TCO electrode. The external quantum efficiency (EQE) measured at 1050 nm is analyzed to further confirm this interpretation (Figure 4g) [55]. The *P*_71.4%_ device exhibits the highest EQE of 59.1%, which agrees with the device performance trends. Despite its reduced optical absorption, the EQE enhancement clearly demonstrates that the FSF layer facilitates superior photocarrier collection through a strong built‐in electric field. Moreover, the *P*_71.4%_ film exhibits the narrowest Raman full width at half maximum (65.83 cm^−1^, Figure S17) and the lowest value of E_U_ (331.1 meV, Figure S18). This confirms that the structurally denser and electronically less disordered amorphous network of this film contributes to efficient carrier transport and minimized recombination loss [56]. This electrical interpretation is further supported by the simulated band diagrams shown in Figure S19. The detailed simulation parameters are listed in Table S2. Compared with the pristine *P*_25%_ structure, the insertion of a heavily doped FSF layer induces a more pronounced front‐side band bending at the FSF/*P*_25%_ interface, thereby forming a local surface field that facilitates electron transport toward the TCO while suppressing hole back‐transfer near the front interface. In particular, the electrostatic modulation is much weaker for the *P*_62.5%_ FSF, whereas the *P*_71.4%_ and *P*_83.3%_ FSF layers generate a stronger front‐surface field. Among them, the *P*_71.4%_ FSF provides the most favorable balance between field strength and barrier selectivity, which is consistent with the superior device performance observed under 1050 nm illumination. These results indicate that the FSF‐induced enhancement originates primarily from improved electrostatic carrier separation and collection efficiency rather than from enhanced optical absorption.

The spectral responsivities of the photodiodes are measured in the 400–1600 nm range to evaluate the wavelength‐dependent photoresponse characteristics (Figure 4h). Compared with the pristine device, the device with the FSF layer exhibits a markedly higher responsivity across the entire wavelength range, which reaches approximately 600 mA/W in the 600–900 nm region. Furthermore, it maintains a superior response even in the NIR‐to‐short‐wave infrared region. In this broadband response, the dominant optical absorber is wavelength dependent: photocarrier generation in the visible region is mainly governed by absorption in n‐a‐Si:H, whereas the NIR response is primarily dominated by Te absorption. Nevertheless, across the full spectral range, carrier separation and collection remain governed by the built‐in field and interfacial energetics of the heterojunction. Therefore, the FSF layer enhances the measured responsivity not by changing the primary absorption mechanism itself, but by improving carrier extraction efficiency after photogeneration. This indicates that the FSF layer effectively improves the photocarrier extraction efficiency across the spectrum by strengthening front‐surface band bending and suppressing interfacial recombination near the TCO/n‐a‐Si:H interface, thereby substantially improving the photoelectric conversion performance. The device with the FSF layer exhibits a stable and repeatable photocurrent switching behavior under various illumination intensities (Figure 4i). Distinct on and off current transitions are observed at each illumination level, indicating reliable photoresponse and efficient carrier transport dynamics. The pronounced current contrast between the illuminated and dark states enables precise signal discrimination. This shows that the integration of the optimized Te/n‐a‐Si:H heterojunction and FSF layer provides a promising platform for developing high‐performance flexible optoelectronic systems [57].

In practical applications, flexible optoelectronic devices are continuously subjected to mechanical deformations such as repeated bending. Therefore, achieving superior electrical performance and outstanding mechanical robustness is essential [2]. As shown in Figure 4j, a flexible photodiode with the FSF layer is subjected to cyclic bending tests at various radii (5, 10, 15, and 20 mm) to assess its mechanical reliability. After 1000 bending cycles, dark current slightly increases and normalized responsivity slightly decreases with the decrease in bending radius (Figure 4k). Nevertheless, the device maintains robust and stable photoresponse without any noticeable degradation. At 4000 bending cycles and a bending radius of 5 mm (Figure 4l), both dark current and normalized responsivity exhibit gradual and continuous variation; however, more than 90% of the initial responsivity is retained. These results demonstrate the simultaneous retention of mechanical flexibility and optoelectronic stability in the Te/n‐a‐Si:H‐based photodetector and highlight its robust structural integrity and strong potential for integration with practical flexible and wearable optoelectronic systems.

## 4 Conclusion

A flexible NIR photodiode was fabricated based on an n‐a‐Si:H/Te heterojunction, in which the microstructure of the charge transport layer and the interfacial energetics were precisely engineered by optimizing the *P* ratio and incorporating an FSF layer. The *P*_25%_ n‐a‐Si:H film exhibited suppressed Si–H_2_ bonding, lower defect density, and improved network ordering, thereby forming an electronically coherent interface with crystalline Te. This refined interface provided a favorable pathway for charge transport while effectively suppressing carrier recombination at the heterojunction. Consequently, the n‐a‐Si:H/Te device showed pronounced diode rectification and strong NIR photoresponse centered at 1050 nm owing to the Te‐dominated optical absorption and efficient carrier separation across the built‐in electric field. The performance was further enhanced by incorporating a heavily doped 10‐nm‐thick FSF layer between the TCO and n‐a‐Si:H, which strengthened band bending and mitigated interfacial recombination losses. The responsivity and detectivity of the device with the FSF layer were 5.1 and 2.6 times higher than those of the pristine device. In addition, the device exhibited broadband spectral response (400–1600 nm), stable on‐ and off‐operation, and over 90% responsivity retention after 4000 bending cycles, thus highlighting its mechanical robustness. These results confirm that the simultaneous control of structural ordering and phosphorus doping in n‐a‐Si:H and field engineering via an FSF layer is an effective strategy for enabling efficient carrier transport and optimizing interfacial energetics in inorganic heterojunction optoelectronics. Thus, this work establishes a versatile material‐to‐device design platform for realizing high‐performance, mechanically resilient, and flexible NIR optoelectronic systems.

## Author Contributions

K.‐J.H. contributed to the experiments, data analysis, and manuscript preparation as the first author; B.‐J.P., H.‐W.J., J.‐W.Y., and Y.‐H.K. contributed to the experiments and data analysis as co‐second authors; W.‐I.P. contributed to experiment management and conceptualization as the corresponding author; S.‐W.C. contributed to conceptualization and manuscript review; J.‐D.K. contributed to experiment management, conceptualization, review, and funding acquisition.

## Conflicts of Interest

The authors declare no conflicts of interest.

## Supporting information




**Supporting File**: advs76332‐sup‐0001‐SuppMat.docx.

## Data Availability

The data that support the findings of this study are available from the corresponding author upon reasonable request.
