# Supplementary material for: Systematic Phosphorus‐Driven Structural and Field Engineering of n‐a‐Si:H for Flexible n‐a‐Si:H/Te Near‐Infrared Photodetectors
Source: Adv Sci (Weinh). 2026 Jul 6;13(53):e76332. doi: 10.1002/advs.76332 (PMC13335606; doi:10.1002/advs.76332)
Supplement: Supplementary file 1 [file ADVS-13-e76332-s001.docx]

**Systematic phosphorus-driven structural and field engineering of n-a-Si:H for flexible n-a-Si:H/Te near-infrared Photodetectors**

*Kyeong-jin Hyun^a,b,#^, Soo-Won Choi^a,#,*^, Byeongjin Park^a,c^, Hee-Won Jang^a,d^, Jongwon Yoon^a^, Yonghun Kim^a^, Woon Ik Park^b,*^, and Jung-Dae Kwon^a,*^*

^a^ Energy & Environment Materials Research Division, Korea Institute of Materials Science, Changwon, Gyeongnam 51508, Republic of Korea

^b^ Department of Materials Science and Engineering, Pukyong National University, Busan 48513, Republic of Korea

^c^ Department of Materials Science and Engineering, Ulsan National Institute of Science and Technology (UNIST), Ulsan 44919, Republic of Korea

^d^ Department of Materials Science and Engineering, Pusan National University, Busan 46241, Republic of Korea

**Figure S1**. Calculated hydrogen concentration and total hydrogen content of n-a-Si:H films derived from FTIR spectra.

**Figure S2**. Absorption coefficient spectra of n-a-Si:H films with varying the phosphine-to-silane ratio (*P*_x%_).

**Figure S3**. Current (I)-voltage (V) characteristics measured under transmission line method (TLM) with varying *P*_x%_ ratio; (a) *P*_50%_, (b) *P*_25%_, (c) *P*_14.3%_, and (d) *P*_9.1%_.

**Figure S4**. Diode characteristics of n-a-Si:H/Te heterojunction flexible device under varying the light intensity; (a) linear and (b) logarithmic scale.

**Figure S5**. Determination of (a, b) optical bandgap (E_g_) using Tauc’s method, (c, d) cutoff and Fermi-edge regions for calculating the work function (Φ) via ultraviolet photoelectron spectroscopy (UPS), and (e, f) valence band maximum positions determined by X-ray photoelectron spectroscopy (XPS); (a, c, e) and (b, d, f) correspond to Te and n-a-Si:H (*P*_25%_), respectively.

**Figure S6**. (a) Transmittance and (b) reflectance spectra of n-a-Si:H (*P*_25%_) and Te films.

**Figure S7**. Current (I)-voltage (V) characteristics of n-a-Si:H films measured under dark and 1050 nm light illumination with varying the *P*_x%_ ratio; (a) *P*_50%_, (b) *P*_25%_, (c)*P*_14.3%_, and (d) *P*_9.1%_.

**Figure S8**. Determination of (a, b, c) cutoff and Fermi-edge regions for calculating the work function (Φ) via UPS, (d, e, f) valence band maximum positions determined by XPS, and (g, h, i) optical bandgap (Eg) using Tauc’s method; (a, d, g), (b, e, h), and (c, f, i) correspond to *P*_50%_, *P*_14.3%_ and *P*_9.1%_ of n-a-Si:H films, respectively.

**Figure S9**. Analysis of energy band diagram of n-a-Si:H/Te heterojunction using AFORS-HET numerical simulation with varying the P ratio of n-a-Si:H.

**Figure S10**. AFORS-HET-simulated built-in potential and average built-in electric field of the post-contact n-a-Si:H/Te heterojunction as a function of the *P* ratio in n-a-Si:H.

**Figure S11**. The trend of contact resistance of n-a-Si:H films with varying the *P* ratio.

**Figure S12**. Fourier-transform infrared (FTIR) spectra of heavily-doped n-a-Si:H films; (a) *P*_62.5%_, (b) *P*_71.4%_, and (c) *P*_83.3%_

**Figure S13**. Dielectric function spectra of pristine (*P*_25%_) and heavily-doped n-a-Si:H films measured using spectroscopic ellipsometer; (a) real (ε_1_) and (b) imaginary (ε_2_).

**Figure S14**. Tauc plot spectra of heavily-doped n-a-Si:H films for determining the E_g_.

**Figure S15**. (a, b, c) Cutoff and Fermi-edge regions for calculating the work function (Φ) via UPS, and (d, e, f) valence band maximum positions determined by XPS; (a, d), (b, e), and (c, f) correspond to *P*_62.5%_, *P*_71.4%_, and *P*_83.3%_, respectively.

**Figure S16**. (a) Transmittance and (b) reflectance spectra of flexible n-a-Si:H/Te heterojunction photodetector incorporating of front-surface field (FSF) layers with varying the heavily-doped *P*_x%_ ratio.

**Figure S17**. Raman spectra of heavily-doped n-a-Si:H films.

**Figure S18**. Logarithmic absorption coefficient spectra of heavily-doped n-a-Si:H films for determining the Urbach energy (E_U_).

**Figure S19**. Analysis of energy band diagram of FSF/n-a-Si:H/Te heterojunction using AFORS-HET numerical simulation with varying the P ratio of FSF layers, compared to pristine (*P*_25%_) heterojunction device.

**Table S1.** … Parameters used in AFORS-HET simulation for n-a-Si:H/Te heterojunction NIR photodetectors.

| Parameter | Te | n-a-Si:H (*P*_50%_) | n-a-Si:H (*P*_25%_) | n-a-Si:H (*P*_14.3%_) | n-a-Si:H (*P*_9.1%_) |
| --- | --- | --- | --- | --- | --- |
| Layer thickness (nm) | 20 | 400 | 400 | 400 | 400 |
| Dielectric constant | 27.5 | 11.9 | 11.9 | 11.9 | 11.9 |
| Electron affinity (eV) | 4.0 | 3.95 | 3.91 | 3.74 | 3.61 |
| Band gap (eV) | 0.88 | 1.90 | 1.94 | 1.91 | 1.92 |
| Optical band gap (eV) | 0.88 | 1.90 | 1.94 | 1.91 | 1.92 |
| Valence band density (cm^-3^) | 2.7ⅹ10^19^ | 2ⅹ10^20^ | 2ⅹ10^20^ | 2ⅹ10^20^ | 2ⅹ10^20^ |
| Conduction band density (cm^-3^) | 2.6ⅹ10^19^ | 2ⅹ10^20^ | 2ⅹ10^20^ | 2ⅹ10^20^ | 2ⅹ10^20^ |
| Hole mobility (cm2V-1s-1) | 400 | 0.01 | 0.01 | 0.01 | 0.01 |
| Electron mobility (cm2V-1s-1) | 100 | 1 | 1 | 1 | 1 |
| Thermal velocity of electron (cm/s) | 1ⅹ10^7^ | 1ⅹ10^7^ | 1ⅹ10^7^ | 1ⅹ10^7^ | 1ⅹ10^7^ |
| Thermal velocity of hole (cm/s) | 1ⅹ10^7^ | 1ⅹ10^7^ | 1ⅹ10^7^ | 1ⅹ10^7^ | 1ⅹ10^7^ |
| Doping concentration (cm^-3^)  (E_C_-E_F_ used in n-a-Si:H) | 1.15ⅹ10^15^ | 0.43 eV | 0.45 eV | 0.52 eV | 0.60 eV |
| Layer density (g/cm^3^) | 6.24 | 2.328 | 2.328 | 2.328 | 2.328 |

**Table S2.** … Parameters used in AFORS-HET simulation for design of FSF layers in energy band diagram.

| Parameter | n-a-Si:H (*P_62.5_*_%_) | n-a-Si:H (*P_71.4_*_%_) | n-a-Si:H (*P_83.3_*_%_) |
| --- | --- | --- | --- |
| Layer thickness (nm) | 400 | 400 | 400 |
| Dielectric constant | 11.9 | 11.9 | 11.9 |
| Electron affinity (eV) | 3.93 | 4.14 | 4.18 |
| Band gap (eV) | 1.90 | 1.93 | 1.87 |
| Optical band gap (eV) | 1.90 | 1.93 | 1.87 |
| Valence band density (cm^-3^) | 2ⅹ10^20^ | 2ⅹ10^20^ | 2ⅹ10^20^ |
| Conduction band density (cm^-3^) | 2ⅹ10^20^ | 2ⅹ10^20^ | 2ⅹ10^20^ |
| Hole mobility (cm2V-1s-1) | 0.01 | 0.01 | 0.01 |
| Electron mobility (cm2V-1s-1) | 1 | 1 | 1 |
| Thermal velocity of electron (cm/s) | 1ⅹ10^7^ | 1ⅹ10^7^ | 1ⅹ10^7^ |
| Thermal velocity of hole (cm/s) | 1ⅹ10^7^ | 1ⅹ10^7^ | 1ⅹ10^7^ |
| Doping concentration (cm^-3^)  (E_C_-E_F_ used in n-a-Si:H) | 0.43 eV | 0.24 eV | 0.22 eV |
| Layer density (g/cm^3^) | 2.328 | 2.328 | 2.328 |
